# Molecular and Histopathological Findings of *Giardia duodenalis* in Rodents and Bats from the Atlantic Forest and Cerrado of Southeastern Brazil

**DOI:** 10.3390/microorganisms14102263

**Published:** 2026-10-05

**Authors:** Marcos Tobias de Santana Miglionico, Bruno Pires Miranda, Sócrates Fraga da Costa Neto, Ricardo Moratelli, João Marcos da Silva Barbosa, Teresa Cristina Bergamo do Bomfim, Helena Lúcia Carneiro Santos

**Affiliations:** 1 Fundação Oswaldo Cruz-Fiocruz, Instituto Oswaldo Cruz, Laboratório de Doenças Parasitárias, Rio de Janeiro 21040-360, Brazil; miglioni@gmail.com (M.T.d.S.M.); brunomiranda7@gmail.com (B.P.M.); 2 Programa de Pós-Graduação “Medicina Tropical”, Instituto Oswaldo Cruz—Fiocruz, Rio de Janeiro 21040-360, Brazil; 3 Fiocruz Mata Atlântica, Fundação Oswaldo Cruz, Rio de Janeiro 22713-560, Brazil; socrates@ioc.fiocruz.br (S.F.d.C.N.); rmoratelli@fiocruz.br (R.M.); 4 Laboratório de Anatomia Patológica Veterinária, Laboratório de Zoonoses, Hospital Municipal Veterinária Jorge Vaitsman, Rio de Janeiro 20941-160, Brazil; jmarcosmedvet@gmail.com; 5 Instituto de Veterinária, Departamento de Parasitologia, Universidade Federal Rural do Rio de Janeiro, Seropédica, Rio de Janeiro 23890-000, Brazil; tcbb@ufrj.br

**Keywords:** *Giardia duodenalis*, β-giardin, assemblage, histopathology, Atlantic Forest, Cerrado, rodents, bats, zoonotic potential

## Abstract

*Giardia duodenalis* is a globally distributed intestinal protozoan infecting humans and animals. This study investigated its occurrence, genetic diversity, and histopathological findings in rodents and bats from the Atlantic Forest and Cerrado of southeastern Brazil. A total of 518 intestinal-content samples, each obtained from a different individual animal, were screened using Sheather’s sugar flotation. Histopathological examination was performed on three tissue sections obtained from the proximal portion of the small intestine. Samples with structures morphologically compatible with *Giardia* cysts were subsequently subjected to nested PCR and partial β-giardin (bg) gene sequencing. Giardia cysts were detected in 13/518 animals (2.5%), including *Oligoryzomys* sp., *O. nigripes*, *Thaptomys nigrita*, *Rattus rattus*, and *Carollia perspicillata.* All 13 microscopy-positive samples were confirmed as *G. duodenalis* by DNA sequencing. Single-locus bg analysis identified 12 isolates as assemblage A, whereas one R. rattus isolate clustered with assemblage E. Because molecular testing was restricted to microscopy-positive samples, the 2.5% estimate represents microscopy-detected occurrence rather than molecular prevalence. Histopathological examination revealed predominantly mild-to-moderate intestinal changes. Structures morphologically compatible with *Giardia* trophozoites were observed in the submucosa of two rodents, although their identity requires specific confirmation. Detection of *G. duodenalis* in *T. nigrita* expands the documented host range, while its detection in *C. perspicillata* extends the documented occurrence of this parasite in this bat host to the Brazilian Atlantic Forest. These findings expand knowledge of *G. duodenalis* genetic circulation in free-ranging small mammals and support integrated surveillance within a One Health framework.

## 1. Introduction

*Giardia duodenalis* (syn. *G. intestinalis* or *G. lamblia*) is a globally distributed protozoan parasite of major public health importance and one of the leading causes of waterborne gastrointestinal disease. It infects humans and a broad diversity of domestic and wild vertebrates, with transmission occurring primarily through the fecal–oral route via the ingestion of environmentally resistant cysts. The prolonged survival of these cysts in water and moist soil facilitates environmental contamination, which plays a central role in the epidemiology and persistence of giardiasis [1,2].

Molecular epidemiological studies have demonstrated that *G. duodenalis* is currently recognized as a species complex divided into eight genetically distinct assemblages (A–H), which differ in host specificity and zoonotic potential [1,2]. Assemblages A and B are responsible for most human infections and exhibit the broadest host ranges, infecting humans, domestic animals, wildlife, and environmental matrices. Subassemblages AI, AII, BIII, and BIV have been frequently reported in epidemiological studies involving humans and animals, whereas AIII has been predominantly associated with wildlife [1,2,3]. In contrast, assemblages C–H are generally considered host-adapted, occurring mainly in canids, livestock, felids, rodents, and marine mammals. However, increasing reports of atypical host infections suggest that host specificity may be more flexible than previously recognized, particularly in anthropized environments.

The Atlantic Forest and Cerrado biomes are among the most biodiverse yet highly threatened ecosystems in Brazil, currently subjected to intense anthropogenic pressure driven by urbanization, agriculture, and habitat fragmentation. The Atlantic Forest has been reduced to a mosaic of forest fragments distributed from Piauí to Rio Grande do Sul, representing approximately 8.5% of the original coverage [4]. Despite extensive environmental degradation, the region is recognized as a biodiversity hotspot characterized by high species richness and endemism [5]. It supports over 320 mammal species, of which approximately 90 are endemic. Many of these mammals serve as reservoirs for parasites with zoonotic potential [6]. Additionally, a large proportion of the Brazilian population is concentrated in these biomes, and disordered urban expansion has continuously increased human–wildlife contact [7,8]. Even with the existence of legally protected areas, including Permanent Preservation Areas (APPs), Environmental Protection Areas (APAs), Conservation Units, and Private Natural Heritage Reserves (RPPNs) within the National System of Conservation Units (SNUC), deforestation and natural resource exploitation remain frequent. Such environmental disturbances can alter host–parasite interactions and facilitate the emergence and circulation of zoonotic pathogens at the human–animal–environment interface [9,10,11,12]. Small mammals, particularly rodents and bats, are important hosts of a wide diversity of pathogens and may contribute to their circulation across natural and human-modified landscapes. Rodents are among the most abundant mammals in these environments, and their ecological plasticity, high population densities, and, in some species, synanthropic behavior increase opportunities for contact with humans and domestic animals. Molecular studies have demonstrated the occurrence of zoonotic and host-associated *G. duodenalis* assemblages in several rodent species, supporting the epidemiological relevance of this mammalian group [13,14]. Bats, in turn, play essential ecological roles as pollinators, seed dispersers, and insect regulators, while their mobility and use of diverse habitats may facilitate exposure to environmentally transmitted parasites. Although their precise role in the epidemiology of giardiasis remains incompletely understood, zoonotic *G. duodenalis* assemblages have been reported in bats from different regions, including the Brazilian Amazon [7,15]. Consequently, rodents and bats may act as wildlife hosts of zoonotic *G. duodenalis* assemblages and potentially contribute to the environmental circulation of the parasite. Despite the ecological and epidemiological relevance of small mammals, information regarding the occurrence, molecular diversity, and pathological significance of *G. duodenalis* infections in these hosts remains limited, particularly in anthropized Atlantic Forest and Cerrado landscapes in Brazil [13,16]. Therefore, this study investigated the occurrence, molecular assemblages, and histopathological alterations associated with *G. duodenalis* infection in free-ranging small mammals from Atlantic Forest fragments in Pedra Branca State Park and Itaguaí, Rio de Janeiro State, and Conceição dos Ouros and Montes Claros, Minas Gerais State. By integrating molecular characterization with histopathological evaluation, this study aimed to provide new insights into the circulation, zoonotic potential, and pathological significance of *G. duodenalis* in wildlife within a One Health framework.

## 2. Materials and Methods

### 2.1. Ethical Statement and Study Area

**Figure 1:**
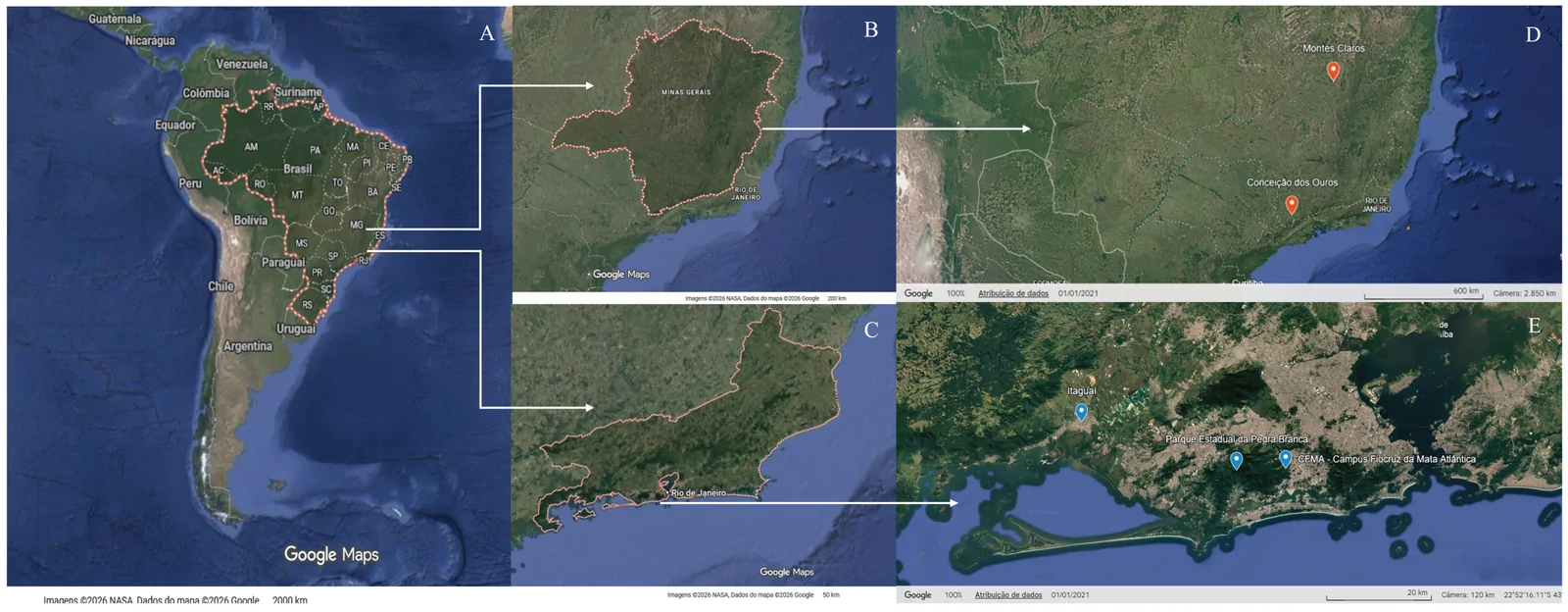
Geographic location of bat and rodent capture sites in regions within the states of Rio de Janeiro and Minas Gerais, Brazil. (**A**) Location of Brazil in South America; (**B**) state of Minas Gerais; (**C**) state of Rio de Janeiro; (**D**) location of collection sites in the state of Minas Gerais; and (**E**) location of collection sites in the state of Rio de Janeiro.

The study protocol was reviewed and approved by the Ethics Committee on Animal Use of the Oswaldo Cruz Foundation, Rio de Janeiro, Brazil (CEUA-IOC, licenses No. LW-39/14, L-036/18), (CEUA-FIOCRUZ, licenses No. LM-6/18, LW-57/19, LW-59/19). Animal capture and sampling were authorized by the Chico Mendes Institute for Biodiversity Conservation (ICMBio; license numbers No. 13373, 14850-1, 19037-1, 69289-1, 59380-3) and by the State Environmental Institute of Rio de Janeiro (INEA; license number No. 020/2011, 012/2021, 007/2020). Field sampling was conducted at five locations in southeastern Brazil: (i) Fiocruz Atlantic Forest Campus (CFMA; 22°56′18″ S 43°24′11″ W) and (ii) Pedra Branca State Park (PEPB; 22°55′57″ S 43°26′34″ W), both located in the municipality of Rio de Janeiro, Rio de Janeiro State, (iii) a rural area in the municipality of Itaguaí, Rio de Janeiro State (22°51′07″ S, 43°46′30″ O), (iv) the municipality of Conceição dos Ouros, Minas Gerais State (MCOU; 22°24′56.42″ S 45°47′53.07″ W), and (v) a Cerrado area in the municipality of Monte Claros, Minas Gerais State (MMCL; 16°42′8.76″ S 43°54′57.98″ W) (Figure 1).

Voucher specimens of rodents and bats were deposited in the Integrated Collection of Wild Mammals and Reservoirs (COLMASTO) at IOC/FIOCRUZ.

### 2.2. Sampling and Material Collection

**Figure 2:**
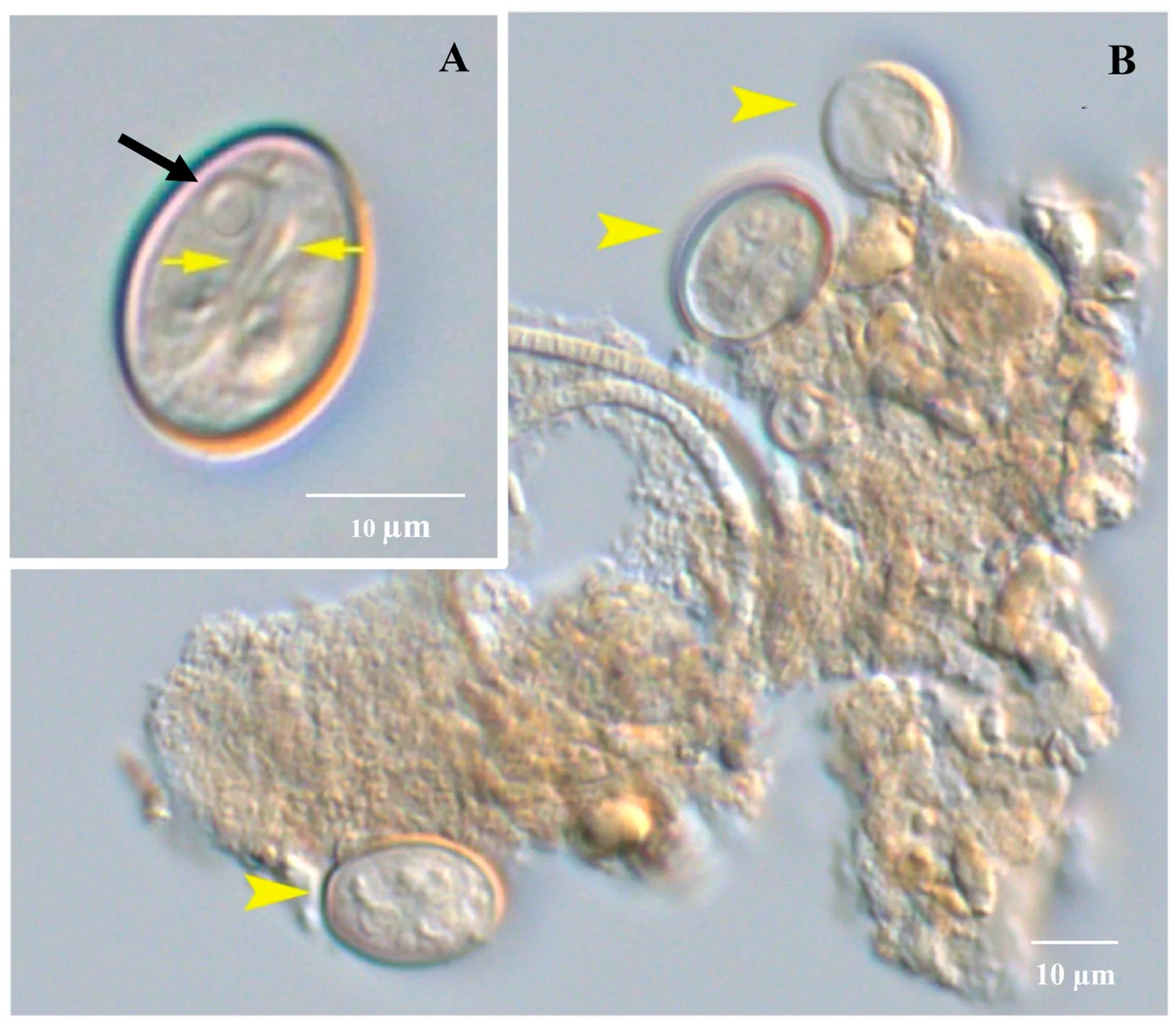
*Giardia duodenalis* detected in small mammals sampled in the Atlantic Forest and Cerrado of southeastern Brazil. Parasitological examination: (**A**) *Giardia* cyst showing a nucleus (black arrow) and axonemes (yellow arrow) and (**B**) multiple *Giardia* cysts (yellow arrows) detected in a fecal sample.

Intestinal-content samples from rodents and bats were collected between August 2019 and September 2022. Following necropsy, intestinal contents were collected individually, transferred to labeled plastic containers, and transported to the laboratory. A total of 518 intestinal-content samples were analyzed, each corresponding to a different individual animal, comprising 258 rodents and 260 bats. Samples were preserved in 2.5% (*w*/*v*) potassium dichromate for two weeks at 23–28 °C. Before coproparasitological examination, potassium dichromate was removed by 10 consecutive washes with 20 mL of phosphate-buffered saline (PBS). At each washing step, samples were centrifuged at 350× *g* for 5 min, the supernatant was discarded, and the sediment was resuspended in fresh PBS. All 518 samples were examined using Sheather’s sugar flotation technique [17] and screened by light microscopy for structures morphologically compatible with *G. duodenalis* cysts (Figure 2). Thirteen samples containing *Giardia* cysts were subsequently subjected to DNA extraction and molecular analysis targeting β-giardin (*bg*) gene.

### 2.3. DNA Extraction, PCR Nested and Phylogenetic Analysis

DNA was extracted from fecal samples that were microscopy-positive for *Giardia* using the QIAamp Fast DNA Stool Mini Kit (QIAGEN, Hilden, Germany), according to the manufacturer’s instructions. DNA extracted from *G. duodenalis* trophozoites was used as a positive amplification control, DNA from *Entamoeba histolytica* was included as a non-target negative amplification control, and water was used as a no-template control (NTC). To minimize cross-contamination during nested PCR, pre-amplification and post-amplification procedures were performed in physically separated work areas using dedicated benches. Filter-barrier pipette tips were used throughout, and amplified PCR products were opened and handled only in the designated post-amplification area.

A partial fragment of the β-giardin (*bg*) gene was amplified by nested PCR. The first amplification round used primers G7 (5′-AAGCCCGACGACCTCACCCGCAGTGC-3′) and G759 (5′-GAGGCCGCCCTGGATCTTCGAGACGAC-3′). The second round used primers BG511F (5′-GAACGAACGAGATCGAGGTCCG-3′) and BG511R (5′-CTCGACGAGCTTCGTGTT-3′), generating an expected amplicon of approximately 511 bp [18]. PCR reaction was performed in a final volume of 50 μL, containing 100 mM of Tris-HCl (pH 9.0), 500 mM of KCl, 2.0 mM of MgCl_2_, 0.2 mM each of dATP, dGTP, dCTP and dTTP, 0.2 μM of primer, 1U of Class Vive Taq DNA polymerase (Neobio, Botucatu SP, BRA), 1% of Triton X-100 and 5 μL of DNA sample. The reactions were performed in a total of 35 cycles using the following conditions: 1st PCR (95 °C for 30 s, 61 °C for 30 s and 72 °C for 60 s) and nested-PCR (95 °C for 30 s, 61 °C for 30 s and 72 °C for 60 s). Reactions began with an initial hot-start step (95 °C for 3 min) and a final extension for 7 min at 72 °C. Obtained amplicons were loaded on 1.5% agarose gels in a Tris-borate–ethylenediaminetetraacetic acid buffer, run at 40 V for 30 min, stained with Nancy-520 and then viewed under UV transillumination. PCR products were purified using the Wizard SV gel and PCR Clean-Up System kit (Promega, Madison, WI, USA), sequenced in both directions using the BigDye Terminator v.3.1 Cycle Sequencing Kit (Applied Biosystems, Foster City, CA, USA), and run on the ABI 3730 Sequencing Platform. The sequences were analyzed and edited using the SeqMan software 6 (DNASTAR software package, DNASTAR Inc., Madison, WI, USA) and compared with previously published sequences using the BLASTN software from the NCBI server (http://www.ncbi.nlm.nih.gov/BLAST, accessed on 15 May 2026). The consensus sequences were compared with reference sequences available in the National Center for Biotechnology Information (NCBI) database using the Basic Local Alignment Search Tool (BLAST) to determine sequence similarity. Additional *G. duodenalis* reference sequences were retrieved from GenBank, and multiple sequence alignments were performed using the Multiple Alignment using Fast Fourier Transform (MAFFT) algorithm (https://mafft.cbrc.jp/alignment/server/, accessed on 15 May 2026). Phylogenetic trees were reconstructed to determine the evolutionary relationships of the isolates, using *Giardia muris* as the outgroup. Two probabilistic phylogenetic approaches were employed: Maximum Likelihood (ML) and Bayesian Inference (BI). The best-fit nucleotide substitution models differed between the phylogenetic approaches. GTR + F + I (general time-reversible model with empirical base frequencies and a proportion of invariant sites) was selected for the Maximum Likelihood (ML) analysis, whereas TIM, with among-site rate heterogeneity modeled using a discrete gamma distribution and four rate categories, was selected for the Bayesian Inference (BI) analysis. All phylogenetic analyses were performed using PhyloSuite v1.2.3. All sequences generated were deposited in the GenBank database under the accession Numbers PZ858567–PZ858579.

### 2.4. Histopathological Analysis of Intestinal Tissues

The proximal portion of the duodenum was collected from all 518 animals submitted to necropsy. A 1 cm duodenal segment from each animal was transversely sectioned at three points, yielding four tissue fragments. The fragments were immediately fixed in 10% Carson’s buffered formalin, routinely processed, embedded in paraffin, sectioned, and stained with hematoxylin and eosin (H&E).

Histological sections from all 518 animals were examined by a veterinary pathologist blinded to the coproparasitological and molecular results. Histopathological evaluation was therefore performed independently of the *Giardia* infection status. The evaluation included mucosal architecture, epithelial integrity, villus and crypt morphology, distribution and cellular composition of inflammatory infiltrates, intraepithelial lymphocytes, edema, vascular congestion, epithelial degeneration and detachment, villous blunting, and alterations in the villus-to-crypt ratio. Structures morphologically compatible with *Giardia* trophozoites were recorded according to their location within the intestinal lumen, adjacent to the epithelial surface, or within deeper tissue layers.

Histopathological changes were graded using a semiquantitative scoring system ranging from 0 to 3, based on inflammatory cellularity, distribution of the inflammatory infiltrate, cellular composition, and the presence of epithelial and architectural alterations (Supplementary Table S1). Score 0 indicated cellularity compatible with the physiological pattern, without evident inflammatory expansion of the lamina propria or relevant epithelial injury. Score 1 indicated a mild increase in the inflammatory infiltrate without relevant structural alterations; score 2 indicated a moderate inflammatory infiltrate with evident expansion of the lamina propria and/or mild epithelial alterations; and score 3 indicated a marked diffuse inflammatory infiltrate associated with cryptitis, crypt abscesses, erosion, ulceration, necrosis, or architectural disorganization. Approximate inflammatory cell counts of 1–50, 51–200, and ≥201 cells were used as auxiliary criteria for scores 1, 2, and 3, respectively, rather than as the sole determinant of histopathological grade [19,20,21].

Low numbers of lymphocytes, plasma cells, macrophages, eosinophils, or mast cells without expansion of the lamina propria were not considered, in isolation, indicative of inflammation. Intraepithelial or intracryptic neutrophils, cryptitis, crypt abscesses, erosion, and ulceration were considered evidence of active inflammation. The distribution of the inflammatory infiltrate was recorded as focal, multifocal, or diffuse, and its cellular composition as lymphoplasmacytic, neutrophilic, eosinophilic, macrophagic, or mixed.

Although all 518 animals underwent blinded histopathological examination, animals without microscopically detected *Giardia* were not used as a formal *Giardia*-negative comparison group because other intestinal protozoa were detected in part of the sampled population.

### 2.5. Statistical Analysis

Microscopy-detected occurrence was calculated as the number of microscopy-positive animals divided by the number examined. Exact 95% confidence intervals (95% CI) were calculated using the Clopper–Pearson method. The occurrence of *G. duodenalis* in rodents and bats was compared using a two-sided Fisher’s exact test. Statistical significance was defined as *p* < 0.05.

## 3. Results

A total of 518 intestinal-content samples from rodents and bats were examined using Sheather’s sugar flotation technique (Figure 2). *Giardia* cysts were detected in 13 samples, corresponding to an overall microscopy-detected occurrence of 2.51% (13/518; exact 95% CI: 1.34–4.25%). Of the 518 animals examined, 268 were sampled in the state of Rio de Janeiro, with eight microscopy-positive individuals (8/268; 2.99%), whereas 250 were sampled in the state of Minas Gerais, with five microscopy-positive individuals (5/250; 2.00%). Regarding biome, 498 animals were sampled in the Atlantic Forest, where all 13 microscopy-positive individuals were detected (13/498; 2.61%), whereas none of the 20 animals sampled in the Cerrado were positive.

**Figure 3:**
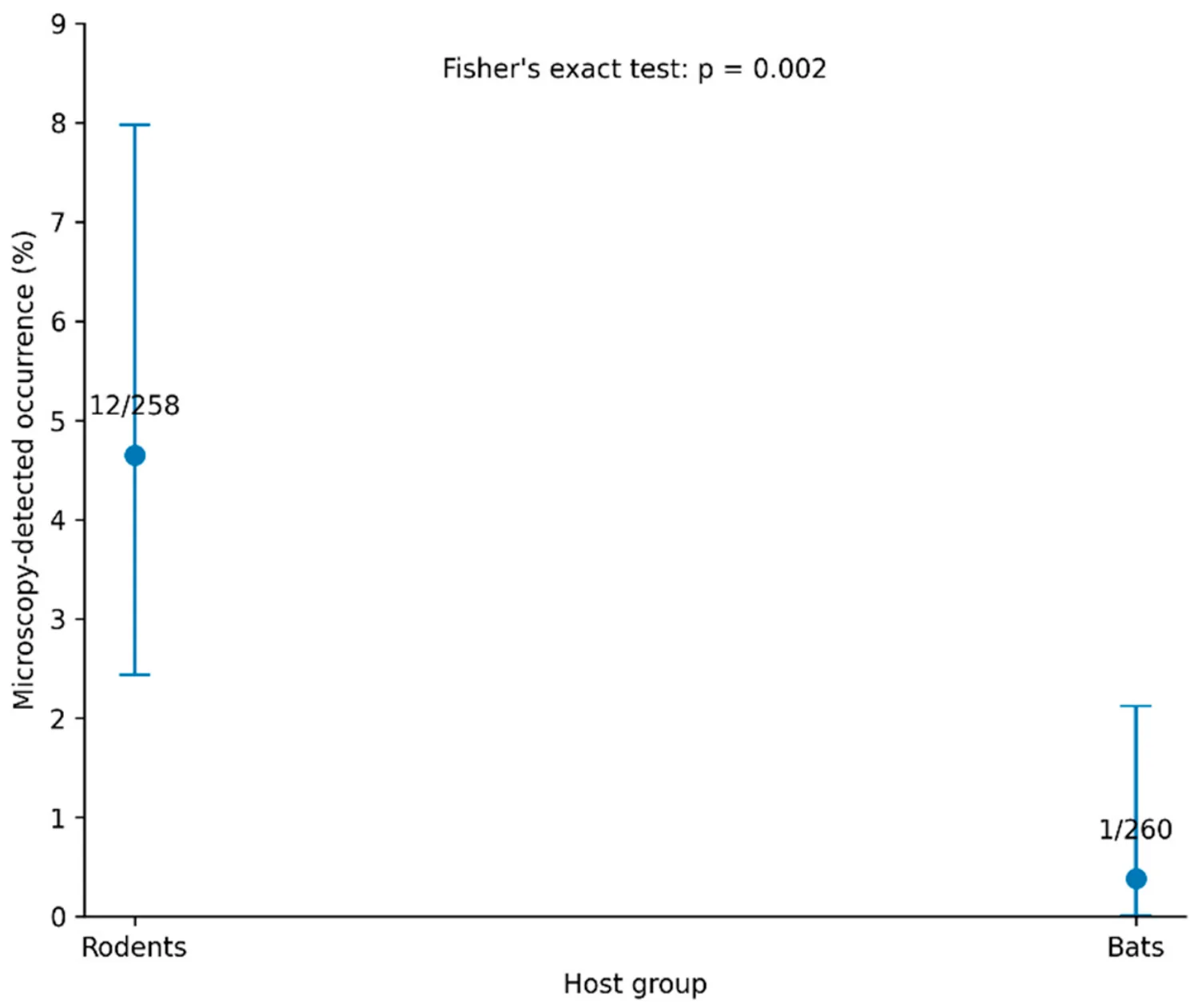
Microscopy-detected occurrence of *Giardia duodenalis* in rodents and bats from southeastern Brazil. Points represent the proportion of microscopy-positive animals, and error bars indicate exact 95% confidence intervals calculated using the Clopper–Pearson method. Microscopy-detected occurrence was significantly higher in rodents (12/258; 4.65%; 95% CI: 2.43–7.98%) than in bats (1/260; 0.38%; 95% CI: 0.01–2.12%) (Fisher’s exact test, *p* = 0.002).

All 13 microscopy-positive samples yielded the expected *β*-giardi gene amplicon of approximately 511 bp by nested PCR and were successfully sequenced, confirming *G. duodenalis* infection. Among rodents, 12 of 258 animals were positive (4.7%; 95% CI: 2.43–7.98%), whereas one of 260 bats was positive (0.4%; 95% CI: 0.01–2.12%), identified as *Carollia perspicillata* (Figure 3).

The difference between rodents and bats was statistically significant (two-sided Fisher’s exact test, *p* = 0.0016). Positive rodents belonged to *Oligoryzomys* sp., *O. nigripes*, *Thaptomys nigrita*, and *Rattus rattus*. The single positive bat was *Carollia perspicillata*. Host-specific results are summarized in Table 1.

All 13 confirmed *G. duodenalis*-positive samples were successfully amplified and partially sequenced at the β-giardin (bg) locus. Comparison with references sequences available in GenBank revealed nucleotide identities ranging from 97.6% to 100%. Based on single-locus β-giardin sequence similarity and phylogenetic clustering, 12 isolates were identified as assemblage A, whereas one isolate from *Rattus rattus* was clustered with reference sequences of assemblage E (Table 2). Because only the *bg* locus was analyzed, no subassemblage-level assignments were made, and the assemblage E result should be regarded as a single-locus finding requiring confirmation with an additional independent locus.

**Figure 4:**
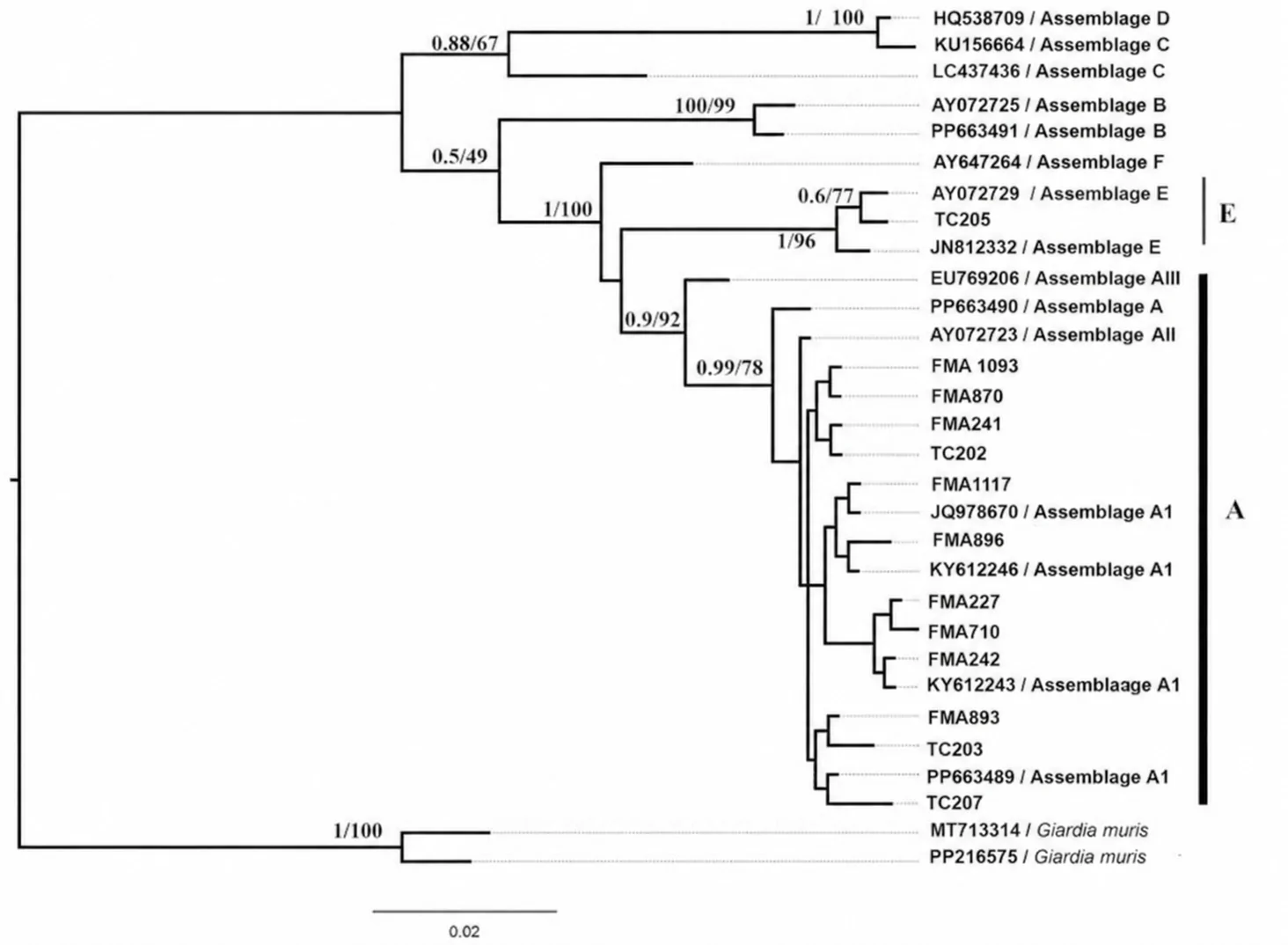
Phylogenetic relationships of *Giardia duodenalis* isolates from rodents and bats based on partial β-giardin (bg) gene sequences. The phylogenetic tree shows the clustering of the isolates characterized in this study within assemblage A and assemblage E, together with reference sequences retrieved from GenBank. Node support values are presented as Bayesian posterior probabilities (BI) followed by Maximum Likelihood bootstrap values (ML) (BI/ML). Bayesian posterior probabilities ≥ 0.5 and ML bootstrap values ≥ 50% are shown at the nodes; branches without displayed support values correspond to Bayesian posterior probabilities < 0.5 and/or ML bootstrap values < 50%. GenBank accession numbers and the corresponding assemblages are indicated for reference sequences. *Giardia muris* was used as the outgroup. The scale bar represents 0.02 nucleotide substitutions per site.

Phylogenetic analyses using Maximum Likelihood and Bayesian Inference showed that 12 of the 13 isolates clustered with assemblage A reference sequences, whereas one isolate clustered within assemblage E (Figure 4). These results represent single-locus phylogenetic classification and should not be interpreted as multilocus genotyping.

**Figure 5:**
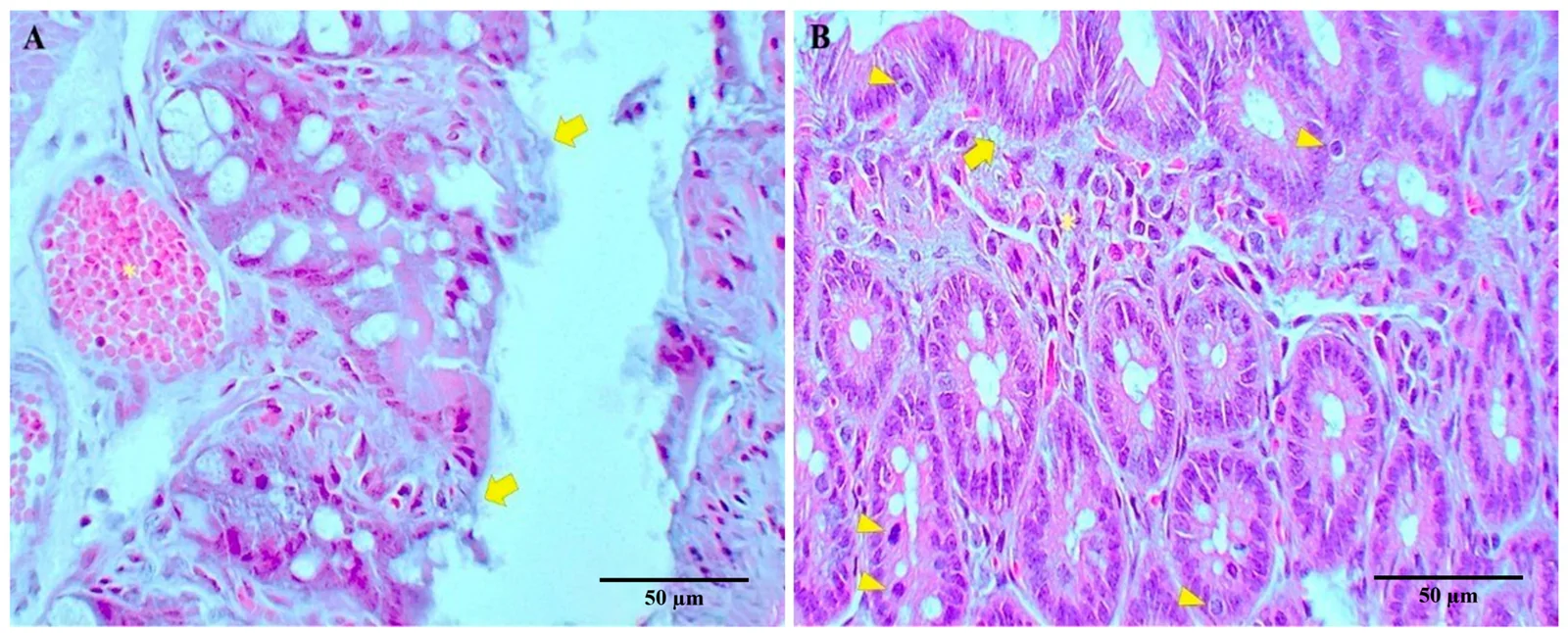
Representative histopathological lesions in the small intestine of a *Giardia duodenalis*-positive animal. (**A**) Villus blunting with focal epithelial erosion (arrows) and submucosal congestion (*). (**B**) Marked villus blunting (arrow), increased intraepithelial lymphocytes (arrow), and lymphoplasmacytic infiltrate with fibrosis of the lamina propria (*). Hematoxylin and eosin (H&E) stain. Scale bars = 50 µm in (**A**,**B**).

Histopathological examination of intestinal tissues from *G. duodenalis*-positive animals revealed predominantly mild-to-moderate inflammatory changes (Supplementary Table S1). The inflammatory infiltrate was mainly composed of lymphocytes and plasma cells, with occasional neutrophils. Increased numbers of intraepithelial lymphocytes and mild edema of the lamina propria were also frequently observed. Epithelial alterations included degeneration and focal epithelial detachment. Architectural changes, including villus blunting, a reduced villus-to-crypt ratio, and mild crypt dilation, were observed in several specimens and were particularly evident in samples FMA241 and FMA242 (Figure 5).

**Figure 6:**
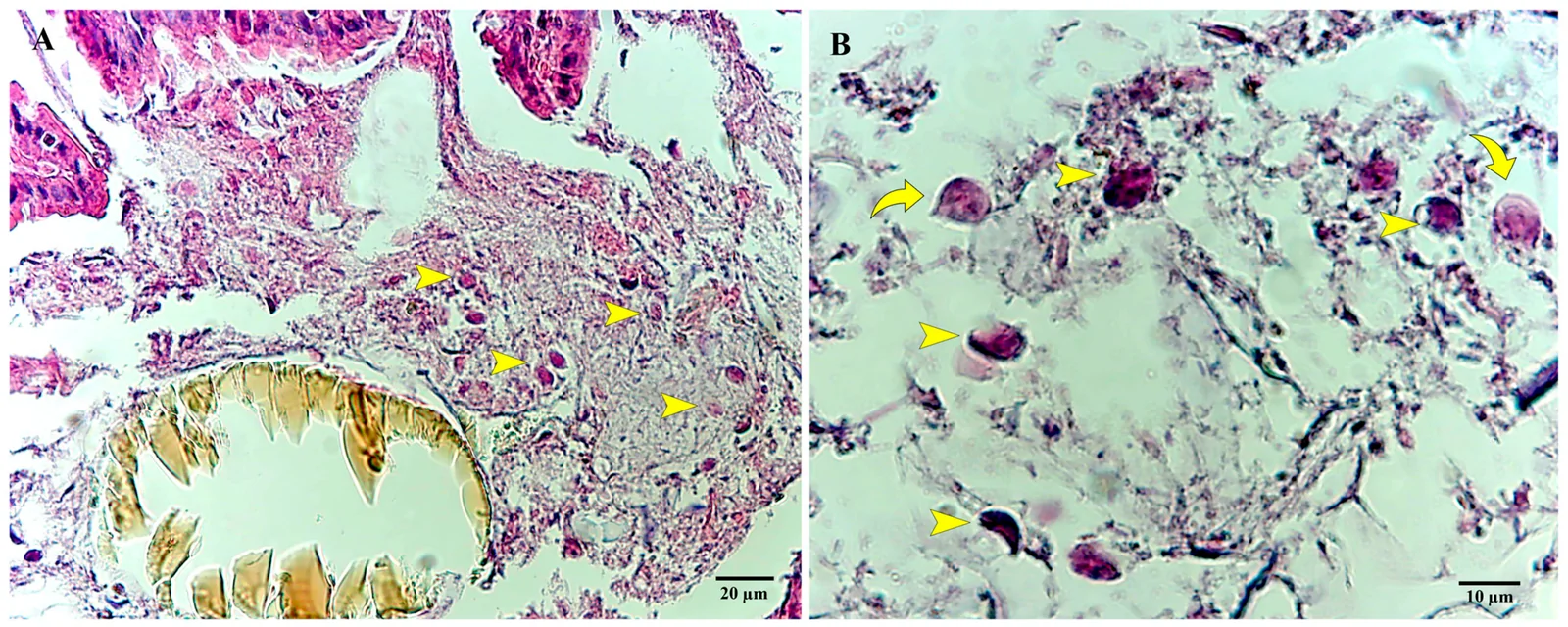
Representative histological sections of the small intestine from a *Giardia duodenalis*-positive animal, showing structures morphologically compatible with *Giardia trophozoites* (arrows) adherent to the apical surface of the intestinal epithelium. Hematoxylin and eosin (HE) stain. Scale bars: (**A**) 20 µm; (**B**) 10 µm.

*G. duodenalis* trophozoites were observed free within the intestinal lumen or closely associated with the epithelial surface (Figure 5). No extensive ulceration or transmural necrosis was observed, and the lesions were predominantly confined to the superficial intestinal mucosa. Notably, in two specimens from *Thaptomys nigrita* and *Oligoryzomys* sp., similar trophozoite-like structures were identified within the intestinal submucosa (Figure 6).

## 4. Discussion

The present study expands current knowledge of *Giardia duodenalis* infection in free-ranging small mammals. The integration of coproparasitological, molecular, and histopathological approaches allowed the characterization of infections in small mammals sampled from natural and anthropogenically influenced sites within the Atlantic Forest and Cerrado biomes of southeastern Brazil. Although the overall prevalence was relatively low (2.5%), the lower sensitivity of a single flotation examination, particularly in low-intensity infections or during intermittent cyst shedding, may have resulted in an underestimation of the actual occurrence of *G. duodenalis* in the sampled populations. Molecular confirmation of all positive samples and the identification of assemblages A and E provided evidence of the occurrence of both zoonotic and host-adapted genotypes in the sampled areas. Furthermore, the association between molecular detection and histopathological findings supports the presence of intestinal infection in the examined animals and strengthens the biological interpretation of the results. Molecular epidemiological studies have demonstrated that markers such as the β-giardin gene are valuable tools for investigating the genetic diversity, host specificity, and transmission patterns of this protozoan parasite [6,7,22].

Rodents accounted for the majority of *G. duodenalis*-positive animals, consistent with previous reports of infection in this mammalian group. Synanthropic species such as *Rattus rattus* are highly adaptable to urban environments, agricultural landscapes, and fragmented forests, increasing opportunities for contact with domestic animals and humans. The detection of *G. duodenalis* in native rodents, including *Oligoryzomys* spp. and *Thaptomys nigrita*, further indicates that infection is not restricted to synanthropic hosts. The occurrence of the parasite in native species from forest remnants suggests that *G. duodenalis* may also be maintained in wild small-mammal populations [14,23,24,25,26,27].

Recent global evidence further demonstrates considerable heterogeneity in the molecular epidemiology of *G. duodenalis* in rodents. A systematic review and meta-analysis reported variation in molecular prevalence and assemblage distribution according to host species, geographic region, and diagnostic approach, with assemblages A–E and G documented in rodents [28]. These findings reinforce the need for caution when comparing estimates among studies and provide a broader context for the predominance of assemblage A observed in the rodents examined here. Moreover, recent systematic reviews and meta-analyses in different animal hosts, including pigs, rabbits, and camelids, have also demonstrated substantial variation in *G. duodenalis* assemblage distribution among host populations and geographic regions [29,30,31]. Collectively, these findings indicate that the detection of a given assemblage in an animal host should not, by itself, be interpreted as evidence of interspecific or zoonotic transmission. Rather, assemblage detection provides evidence of genetic circulation within the sampled host population and geographic context. Although *G. duodenalis* was detected in only a single bat, the finding is noteworthy considering the ecological characteristics of this mammalian group. High mobility, colonial behavior, and movement among forest fragments may increase opportunities for the dispersal of fecal–oral pathogens. Recent reviews have shown that several bat species can harbor zoonotic assemblages of *Giardia* spp., although their role in the epidemiology of giardiasis remains poorly understood [15]. In Brazil, Reis and colleagues [7] identified assemblages A and B in Amazonian bats, indicating the occurrence of potentially zoonotic genotypes in wild chiropteran populations. In this context, the detection of assemblage A in *Carollia perspicillata* expands current knowledge of *G. duodenalis* infection in Brazilian bats and highlights the value of including chiropteran species in studies investigating the diversity and host range of *Giardia* spp. at wildlife–domestic animal–human interfaces. One of the principal findings of this study was the identification of *G. duodenalis* assemblages A and E in free-ranging small mammals. The detection of both zoonotic and host-adapted assemblages highlights the genetic diversity of this parasite in the sampled areas and underscores the importance of molecular epidemiology for investigating potential transmission patterns [13,26,27]. Assemblage A is widely distributed among humans, domestic animals, and wildlife and is recognized for its broad host ranges and zoonotic potential [27,32]. The occurrence of assemblage A in *Rattus rattus*, *Oligoryzomys* spp., *Thaptomys nigrita*, and *Carollia perspicillata* indicates its presence across taxonomically and ecologically distinct small-mammal hosts. This distribution may reflect exposure to shared environmental sources of infection and suggest potential transmission interfaces among wildlife, domestic animals, and human populations. The detection of assemblage E in *R. rattus* deserves particular attention, as this assemblage has traditionally been associated with livestock, particularly cattle, sheep, and goats. However, recent reports of assemblage E in wildlife and, occasionally, in humans suggest a broader host range than previously recognized. Collectively, the occurrence of assemblages A and E in small mammals from anthropogenically influenced areas highlights the presence of both zoonotic and host-adapted genotypes in these environments. These findings reinforce the importance of molecular surveillance for improving the understanding of *G. duodenalis* transmission dynamics at wildlife–domestic animal–human interfaces [1,2]. The integration of molecular characterization with histopathological evaluation represents a major strength of this study, allowing genotypic data to be interpreted alongside tissue alterations in naturally infected hosts. Histopathological changes were predominantly mild-to-moderate and included lymphoplasmacytic enteritis, increased numbers of intraepithelial lymphocytes, mild edema of the lamina propria, focal epithelial alterations, and villus blunting. These findings are consistent with the classical pattern of intestinal lesions associated with G. duodenalis infection, in which trophozoites typically remain attached to the intestinal epithelium and pathogenic effects are primarily associated with epithelial dysfunction rather than extensive tissue destruction [33,34]. The absence of ulceration, necrosis, or marked disruption of the intestinal architecture further supports the predominantly mild nature of the lesions observed.

Previous studies have demonstrated that giardiasis may result in partial villus atrophy, increased numbers of intraepithelial lymphocytes, and impaired intestinal absorption even in the absence of extensive mucosal damage [34,35,36,37]. These observations are consistent with the present findings, in which subtle structural alterations occurred alongside mild-to-moderate mucosal inflammation. Experimental and clinical evidence further indicates that *Giardia* infection can disrupt epithelial barrier integrity through alterations in intercellular tight junctions, increased intestinal permeability, and enterocyte apoptosis, involving both host immune responses and parasite-derived factors [34,38]. Although hematoxylin and eosin staining does not permit direct assessment of these molecular mechanisms, the focal epithelial detachment and villus blunting observed in the present study are compatible with epithelial alterations previously associated with *Giardia* infection.

The integration of molecular characterization with histopathological evaluation represents a major strength of this study, allowing genotypic data to be interpreted alongside tissue alterations in naturally infected hosts. Histopathological changes were predominantly mild-to-moderate and included lymphoplasmacytic enteritis, increased numbers of intraepithelial lymphocytes, mild edema of the lamina propria, focal epithelial alterations, and villus blunting. These findings are consistent with the classical pattern of intestinal lesions associated with *Giardia duodenalis* infection, in which trophozoites typically colonize the intestinal lumen and remain attached to the epithelial surface, with functional alterations often being more prominent than extensive tissue destruction [37,39]. The absence of ulceration, necrosis, or marked disruption of the intestinal architecture further supports the predominantly mild nature of the lesions observed.

Previous studies have demonstrated that giardiasis may result in partial villus atrophy, increased numbers of intraepithelial lymphocytes, and impaired intestinal absorption even in the absence of extensive mucosal damage [34,35,36,37]. The pathogenesis of giardiasis has also been associated with disruption of epithelial barrier integrity, including alterations in intercellular tight junctions, increased intestinal permeability, and enterocyte apoptosis, involving both host immune responses and parasite-derived factors [34,38]. More recently, Hagen et al. (2025) provided experimental evidence that trophozoite attachment itself contributes to epithelial barrier disruption. Using a human gastrointestinal organoid model, the authors demonstrated that the characteristic domed architecture of the ventral disc is essential for effective attachment and that mutants with defective disc architecture exhibited a reduced ability to disrupt the epithelial barrier [40]. These findings provide a mechanistic basis for understanding how trophozoite attachment may contribute to epithelial dysfunction. Although hematoxylin and eosin staining does not permit direct assessment of these cellular and molecular mechanisms, the focal epithelial detachment and villus blunting observed in the present study are compatible with epithelial alterations previously associated with *Giardia* infection.

Giardiasis has traditionally been regarded as a non-invasive infection because *G. duodenalis* trophozoites typically remain within the intestinal lumen and attach to the apical surface of enterocytes through the ventral adhesive disc. However, occasional observations have challenged the concept of *Giardia* as a strictly luminal parasite [41]. Early histological studies documented trophozoites within the intestinal mucosa of infected patients, while subsequent immunoperoxidase analyses detected *Giardia* in the human jejunal mucosa, providing further evidence that trophozoites may occasionally occur beyond their usual luminal location [35,42]. More recent evidence from an in vivo experimental model demonstrated *G.-duodenalis* trophozoites crossing the intestinal epithelium and reaching the lamina propria and submucosa [43]. Together, these observations suggest that trophozoite localization beyond the epithelial surface may occur under particular host–parasite conditions, although this remains an uncommon phenomenon.

In the present study, trophozoites morphologically compatible with *Giardia* were observed within the intestinal submucosa of two naturally infected rodents, *Thaptomys nigrita* and *Oligoryzomys* sp., both sampled in Minas Gerais and molecularly characterized as *G. duodenalis* assemblage A. This finding is particularly noteworthy because previous evidence of trophozoites in deeper intestinal tissues has been limited to occasional observations in humans and experimental models. The detection in two naturally infected wild rodents indicates that localization beyond the intestinal epithelium may also occur under natural conditions. Although both animals harbored assemblage A, the available data do not support an association between this genotype and submucosal localization. Furthermore, histopathological examination alone cannot determine whether the presence of trophozoites in the submucosa resulted from active tissue invasion or from translocation associated with disruption of the epithelial barrier. The latter represents a biologically plausible mechanism considering recent evidence that ventral disc-mediated attachment contributes to epithelial barrier disruption [40]; however, barrier disruption itself should not be interpreted as evidence of active parasite invasion.

The presence of trophozoites in the submucosa of two naturally infected rodents represents an unusual histopathological finding. However, the frequency and biological significance of this phenomenon remain unclear, particularly because few studies have systematically investigated the histopathological features of *G. duodenalis* infection in naturally infected wildlife. Consequently, the limited number of reports of trophozoites in deeper intestinal tissues may reflect, at least in part, the scarcity of histopathological investigations rather than the true rarity of this phenomenon. Specific immunohistochemical studies are needed to confirm the presence of *Giardia* trophozoites within the submucosa and to better characterize their tissue localization. Further studies involving naturally infected hosts are also necessary to clarify the mechanisms and biological significance of trophozoite localization beyond the intestinal epithelium.

From a One Health perspective, the detection of *G. duodenalis* in free-ranging small mammals, including zoonotic and host-adapted assemblages, highlights the relevance of wildlife in studies investigating the ecology of this parasite at wildlife–domestic animal–human interfaces. This aspect is particularly important in landscapes where habitat fragmentation and anthropogenic activities increase opportunities for contact among different host populations and shared environmental resources. The combination of molecular characterization and histopathological evidence of intestinal infection further emphasizes the value of integrating wildlife into epidemiological studies of protozoan parasites of medical and veterinary significance.

This study has some limitations. Molecular characterization was based exclusively on the β-giardin gene, and the relatively small number of positive samples limited more detailed analyses of ecological and host-related factors associated with infection. In addition, the cross-sectional design does not allow assessment of infection persistence, cyst-shedding dynamics, or transmission among host species. Nevertheless, the integration of coproparasitological examination, nested PCR, DNA sequencing, and histopathological analysis provided complementary evidence of *G. duodenalis* infection and genetic diversity in free-ranging small mammals from the sampled areas. These findings expand current knowledge of the occurrence of *G. duodenalis* in wildlife and reinforce the importance of multidisciplinary approaches for investigating its ecology and potential transmission at wildlife–domestic animal–human interfaces.

## 5. Conclusions

Free-ranging rodents and bats from the sampled areas harbored both zoonotic and host-adapted assemblages of *Giardia duodenalis assemblages* A and E. Molecular and histopathological findings confirmed intestinal infection and revealed an unusual submucosal localization of trophozoites in naturally infected rodents. The first detection of *G. duodenalis* in *Thaptomys nigrita* and *Carollia perspicillata* expands the known host range of this protozoan in Brazil. Further studies using multilocus genotyping and parasite-specific tissue-based methods are needed to refine assemblage characterization and clarify the biological significance of the histopathological findings within a One Health framework.

## Figures and Tables

**Table 1:** Microscopy-detected occurrence of *Giardia duodenalis* by host taxonomic group.

Order	Family	Host Species	Positive/Examined, n/N; Occurrence % (95% CI)
Rodentia	Cricetidae	*Akodon* sp.	0/193
		*Calomys* sp.	0/1
		*Oligoryzomys* sp.	3/9 (33.3%; 7.5–70.1)
		*Oligoryzomys nigripes*	3/3 (100%; 29.2–100)
		*Thaptomys nigrita*	1/6 (16.7%; 0.4–64.1)
	Muridae	*Rattus norvegicus*	0/2
		*Rattus rattus*	5/44 (11.4%; 3.8–24.6)
Total Rodentia			12/258 (4.7%; 2.4–8.0)
Chiroptera	Emballonuridae	*Peropteryx macrotis*	0/3
	Molossidae	*Eumops* sp.	0/1
		*Molossops temminckii*	0/1
		*Molossus molossus*	0/5
	Phyllostomidae	*Anoura caudifer*	0/5
		*Anoura geoffroyi*	0/6
		*Artibeus fimbriatus*	0/6
		*Artibeus lituratus*	0/45
		*Artibeus obscurus*	0/5
		*Artibeus planirostris*	0/1
		*Carollia brevicauda*	0/4
		*Carollia perspicillata*	1/86 (1.2%; 0.03–6.3)
		*Chiroderma villosum*	0/1
		*Desmodus rotundus*	0/15
		*Glossophaga soricina*	0/6
		*Lonchophylla* sp.	0/1
		*Micronycteris macrotis*	0/3
		*Micronycteris minuta*	0/1
		*Phyllostomus hastatus*	0/3
		*Platyrrhinus lineatus*	0/15
		*Platyrrhinus recifinus*	0/5
		*Sturnira lilium*	0/18
		*Sturnira tildae*	0/1
		*Vampyressa pusilla*	0/4
	Vespertilionidae	*Eptesicus brasiliense*	0/1
		*Eptesicus diminutus*	0/1
		*Eptesicus furinalis*	0/2
		*Myotis* cf. *nigricans*	0/6
		*Myotis lavali*	0/1
		*Myotis nigricans*	0/2
		*Myotis riparius*	0/6
Total Chiroptera			1/260 (0.4%; 0.01–2.1)
Total			13/518 (2.5%; 1.3–4.3)

**Table 2:** Molecular characterization of *Giardia duodenalis* isolates from rodents and bats based on partial β-giardin (bg) sequences.

Isolate Code	Host	Location	Assemblage	Identity (%)/GenBank Reference
Trz02	*Rattus rattus*	RJ	A	(98.94%) KY658181.1
Trz03	*Rattus rattus*	RJ	A	(98.79%) EU274394.1
Trz05	*Rattus rattus*	RJ	E	(99.14%) MN174850.1
Trz07	*Rattus rattus*	RJ	A	(97.60%) KP756607.1
FMA227	*Oligoryzomys* sp.	MG	A	(100%) PV061456.1
FMA241	*Thaptomys nigrita*	MG	A	(99.74%) PV061456.1
FMA242	*Oligoryzomys* sp.	MG	A	(99.71%) PV865535.1
FMA710	*Rattus rattus*	RJ	A	(99.77%) KP756607.1
FMA870	*Oligoryzomys nigripes*	MG	A	(100%) OM115998.1
FMA893	*Oligoryzomys nigripes*	MG	A	(100%) MK452850.1
FMA986	*Carollia perspicillata*	RJ	A	(98.72%) KM926503.1
FMA1093	*Oligoryzomys* sp.	RJ	A	(100%) MK452859.1
FMA1117	*Oligoryzomys nigripes*	RJ	A	(99.32%) JQ978657.1

Note: Assemblage assignment was based on sequence identity at the partial *bg* locus and phylogenetic clustering. Subssemblage-level assignments were not made.

## Data Availability

The nucleotide sequences reported in this paper were deposited in GenBank under PZ858567–PZ858579.

## Supplementary Materials

The following supporting information can be downloaded at https://www.mdpi.com/article/10.3390/microorganisms14102263/s1, Table S1: Individual histopathological findings in Giardia duodenalis microscopy-positive animals.
