# Supplementary material for: Molecular and Histopathological Findings of Giardia duodenalis in Rodents and Bats from the Atlantic Forest and Cerrado of Southeastern Brazil
Source: Microorganisms. 2026 Oct 5;14(10):2263. doi: 10.3390/microorganisms14102263 (PMC13707752; doi:10.3390/microorganisms14102263)
Supplement: Supplementary file 1 [file microorganisms-14-02263-s001.zip › microorganisms-4547800-supplementary.pdf]

Supplementary Table S1: Individual histopathological findings in *Giardia duodenalis* microscopy-positive animals.

Host Species	Epithelial detachment	Epithelial cell degeneration	Erosion	Villous blunting	Crypt dilation	Adaptive changes	Distribution of inflammation	Inflammatory cell composition	Inflammation score
Oligoryzomys sp	Absent	Present	Absent	Absent	Absent	Absent	Diffuse	Lymphoplasmacytic	1
Thaptomys nigrita	Present	Present	Present	Moderate	Present	Absent	Multifocal	Mixed, predominantly lymphocytic	2
Oligoryzomys sp	Present	Absent	Absent	Mild	Absent	Hyperplasia	Diffuse	Lymphoplasmacytic	2
Rattus rattus	Absent	Absent	Absent	Absent	Absent	Hyperplasia	Diffuse	Lymphoplasmacytic	1
Oligoryzomys nigripes	Absent	Absent	Absent	Absent	Absent	Absent	Diffuse	Lymphoplasmacytic	1
Oligoryzomys nigripes	Present	Present	Present	Mild	Absent	Absent	Multifocal	Lymphoplasmacytic	2
Carollia perspicillata	Absent	Absent	Absent	Moderate	Absent	Hyperplasia	Diffuse	Lymphoplasmacytic	2
Oligoryzomys sp	Absent	Present	Absent	Absent	Present	Hyperplasia	Diffuse	Lymphoplasmacytic	1
Oligoryzomys nigripes	Absent	Present	Absent	Absent	Absent	Absent	Multifocal	Lymphoplasmacytic	1
Rattus rattus	Absent	Present	Absent	Absent	Absent	Hyperplasia	Diffuse	Lymphoplasmacytic	1
Rattus rattus	Absent	Present	Absent	Mild	Absent	Hyperplasia	Diffuse	Lymphoplasmacytic	1
Rattus rattus	Present	Present	Absent	Moderate	Absent	Absent	Multifocal	Lymphoplasmacytic	2
Rattus rattus	Absent	Present	Absent	Absent	Present	Hyperplasia	Diffuse	Lymphoplasmacytic	1
